# More flexibility for code generation with GeNN v2.1

**DOI:** 10.1186/1471-2202-16-S1-P291

**Published:** 2015-12-18

**Authors:** Thomas Nowotny, James Turner, Esin Yavuz

**Affiliations:** 1 CCNR, School of Engineering and Informatics, University of Sussex, Falmer, Brighton BN1 9QJ, UK

## Background

GeNN (GPU enhanced Neuronal Networks) [1,2] is a software framework that was designed to facilitate the use of GPUs (Graphics Processing Units) for the simulation of spiking neuronal networks. It is built on top of the CUDA (Common Unified Device Architecture) [3] application programming interface provided by NVIDIA Corporation and is entirely based on code generation: Users provide a compact description of a spiking neuronal network model and GeNN generates CUDA and C++ code to simulate it, also taking into account the specifics of the GPU hardware detected at compile time.

## Methods

In this contribution we describe novel work on GeNN, which has transformed it to a yet more flexible tool for facilitating the use of GPUs for simulations accelerated by GPUs. The main innovations involve replacing previous fixed templates for synapse dynamics and learning models by user-definable code snippets, so allowing redefinition of virtually every dynamic element of a neural network simulation. This transition has also enabled the completion of the Brian2 to GeNN and SpineML to GeNN interfaces [4].

## Results

GeNN now allows the free definition of all four, neuron dynamics, neuron threshold conditions, synapse dynamics and connection weight dynamics (learning). The desired behavior is encoded in code snippets that contain C++ compatible code that describes the operations that are necessary to complete one time step. Table 1 summarizes the available code slots and their function.

**Table 1:** Summary of code slots available in GeNN for user-defined models

Element	Snippet	Deployment and Function
Neuron	simCode	Main time step update of the neuron dynamics

	thresholdConditionCode	A Boolean expression defining when spikes occur, checked every time step

	resetCode	The code that defines a change in neuron variables, employed when a spike occurs

Synapse	simCode	Code that describes the synapse update after a pre-synaptic spike

	simCodeEvnt	Code that describes the synapse update after a pre-synaptic spike event

	simLearnPost	Code for the synaptic update triggered by a post-synaptic spike

	eventThreshold	A Boolean expression that defines synaptic events

	synapseDynamics	Update code for internal synapse dynamics applied every time step independent of spiking

Post-Synapse	postSyntoCurrent	Code that describes the transformation of synaptic variables into a post-synaptic current

	postSynDecay	Code that describes the shared dynamics of summed synaptic activation, typically decay

Other improvements in GeNN 2.1 include an improved CUDA block size estimation algorithm, access to pre- and post-synaptic variables in synaptic models, and a number of bug fixes.

## Conclusion

GeNN has reached level of stability where it should be of increasing use to the wider computational neuroscience community, in particular with the completion of its interfaces to other simulators.
